# Low Light Availability Alters Root Exudation and Reduces Putative Beneficial Microorganisms in Seagrass Roots

**DOI:** 10.3389/fmicb.2017.02667

**Published:** 2018-01-11

**Authors:** Belinda C. Martin, Deirdre Gleeson, John Statton, Andre R. Siebers, Pauline Grierson, Megan H. Ryan, Gary A. Kendrick

**Affiliations:** ^1^School of Biological Sciences, The University of Western Australia, Crawley, WA, Australia; ^2^UWA Oceans Institute, The University of Western Australia, Crawley, WA, Australia; ^3^School of Agriculture and Environment, The University of Western Australia, Crawley, WA, Australia; ^4^Western Australian Marine Science Institution, Perth, WA, Australia; ^5^West Australian Biogeochemistry Centre, School of Biological Sciences, The University of Western Australia, Crawley, WA, Australia

**Keywords:** root microbiome, seagrass, exudation, light, dredging, *Sulfurimonas*, 16S rDNA, PARAFAC-EEM

## Abstract

Seagrass roots host a diverse microbiome that is critical for plant growth and health. Composition of microbial communities can be regulated in part by root exudates, but the specifics of these interactions in seagrass rhizospheres are still largely unknown. As light availability controls primary productivity, reduced light may impact root exudation and consequently the composition of the root microbiome. Hence, we analyzed the influence of light availability on root exudation and community structure of the root microbiome of three co-occurring seagrass species, *Halophila ovalis, Halodule uninervis* and *Cymodocea serrulata*. Plants were grown under four light treatments in mesocosms for 2 weeks; control (100% surface irradiance (SI), medium (40% SI), low (20% SI) and fluctuating light (10 days 20% and 4 days 100%). 16S rDNA amplicon sequencing revealed that microbial diversity, composition and predicted function were strongly influenced by the presence of seagrass roots, such that root microbiomes were unique to each seagrass species. Reduced light availability altered seagrass root exudation, as characterized using fluorescence spectroscopy, and altered the composition of seagrass root microbiomes with a reduction in abundance of potentially beneficial microorganisms. Overall, this study highlights the potential for above-ground light reduction to invoke a cascade of changes from alterations in root exudation to a reduction in putative beneficial microorganisms and, ultimately, confirms the importance of the seagrass root environment – a critical, but often overlooked space.

## Introduction

It has long been established that roots of terrestrial plants are colonized by a diverse assemblage of microbes that collectively function as a ‘microbiome.’ These microbiomes are critical for plant growth and health via their influence on biogeochemical cycling and nutrient acquisition, induction of host defense to pathogens and disease and production of plant growth regulators (see reviews by Reinhold-Hurek et al. (2015) and Alegria Terrazas et al. (2016). Seagrasses, having evolved from land plants, are also colonized by a diverse microbial community that are believed to carry out metabolic functions important for their hosts including nitrogen fixation (Bagwell et al., 2002; Garcias-Bonet et al., 2016), sulfate reduction and oxidation (Küsel et al., 2006), phosphate solubilisation (Ghosh et al., 2012) and nutrient turnover (Duarte et al., 2005; Trevathan-Tackett et al., 2017). However, we are still in the early stages of understanding seagrass–microbe interactions; consequently, whole-organism (seagrass + microbiome) adaptability, metabolic diversity and, therefore, resilience of the seagrass and microbiome to environmental change remains largely unexplored.

Seagrass meadows provide a multitude of beneficial ecosystem services including, habitat structure, carbon sequestration, primary food sources and protection from coastal erosion (Orth et al., 2006). Because seagrasses have a relatively high requirement for light (minimal 11% surface irradiance versus 1% for phytoplankton), they are restricted to growing in shallow coastal environments, which also makes them vulnerable to human and natural activities that can reduce light penetration (e.g., eutrophication, dredging and cyclones) (Orth et al., 2006; Ralph et al., 2007; Waycott et al., 2009). A reduction in light availability reduces photosynthetic performance and therefore impacts negatively on growth, productivity and overall survival of the plant (Biber et al., 2009; Yaakub et al., 2014). Seagrasses avoid negative impacts from short-term light reductions by modifications to their physiology and metabolism; thus, light reduction of more than 1 month may be required to affect growth (Longstaff and Dennison, 1999; Ralph et al., 2007; McMahon et al., 2013). In contrast, microbial communities can respond rapidly to disturbance (Allison and Martiny, 2008) and therefore monitoring their composition could prove an effective early indicator of environmental fluctuations and ecosystem change.

Composition of root-associated microbial communities is controlled by factors at several scales, including those arising from interactions with other microbes (e.g., competition, cross-feeding) as well as regulated at an environmental (e.g., pH, temperature) or host level (plant) (Wagner et al., 2016; Widder et al., 2016; Wemheuer et al., 2017). One of the most important ways in which microbial composition can be regulated from the host level in terrestrial plants is via the exudation of compounds from plant roots. Exudates can shape communities that are specific to the plant species or even plant genotype by selectively stimulating or inhibiting particular microbial populations (Haichar et al., 2012; Chaparro et al., 2014; Alegria Terrazas et al., 2016; Kawasaki et al., 2016; Martin et al., 2016). This root exudate–microbe interaction has been demonstrated to some extent for seagrass species. For example, bacteria isolated from the roots of the seagrasses *Zostera marina* and *Halodule wrightii* showed positive chemotactic responses and preferential substrate utilization to root exudates and root extracts (Wood and Hayasaka, 1981; Kilminster and Garland, 2009). Other studies utilizing ^13^C or ^14^C labeling have directly traced the flow of carbon from seagrasses into the sediment bacteria (Moriarty et al., 1986; Holmer et al., 2001; Kaldy et al., 2006). Given the importance of root microbiomes to host plant health, and ultimately ecosystem function, there is a need to better understand the controls and drivers of microbial compositions in seagrass systems.

Currently, our knowledge of seagrass root exudation rates and composition is limited to a few studies, most from several decades ago (Brylinsky, 1977; Penhale and Smith, 1977; Wetzel and Penhale, 1979; Wood and Hayasaka, 1981; Moriarty et al., 1986; Holmer et al., 2001). These studies highlight the diversity in root exudation patterns (composition and concentration) among different seagrass species. It has also been shown that seagrass metabolism shifts under anoxia (Hasler-Sheetal et al., 2015), which can lead to increased exudation of carbon from roots as fermentation products (Smith et al., 1988). The species-specific nature of root exudation and the change in exudation in reduced light could in turn influence the composition of the root microbiome. However, this top-down effect on the microbiome has not yet been investigated.

The aim of this research was to assess the impact of light reduction on root exudation and the root microbiome of three tropical seagrasses *Halophila ovalis, Halodule uninervis* and *Cymodocea serrulata*. These three species commonly grow in mixed meadows in the northwest of Western Australia. In a previous study we found that root exudation in these three species was increased when light availability was reduced in a fluctuating pattern (Martin et al., 2017). In the present work, we test if the impact of light reduction also affects the root microbiome of these seagrass species through alterations in root exudation and if any variation in the root microbiome among the seagrass species is related to variation in exudation profiles. We selected these three seagrass species as they all have relatively fast growth rates and are quick to respond to environmental disturbance compared to larger seagrass species (Kilminster et al., 2015). We addressed three hypotheses: (i) the composition and predicted function of the root microbiome will differ among seagrass species and also be distinct from the surrounding sediment, (ii) root exudation will be increased by low and/or fluctuating light availability, and (iii) microbial composition and predicted function will be influenced by light availability, but in a seagrass species-specific manner.

## Materials and Methods

### Site Description and Seagrass Collection

Seagrass species were collected from Useless Loop in Shark Bay, Western Australia, in May 2015 (26°07′S 113°24′E). Shark Bay is a subtropical marine embayment that is characterized by phosphorus-limited carbonate sediments and a hyper-salinity gradient extending from north (∼36 psu) to south (50–60 psu) (Kendrick et al., 2012). Ramets with apical shoots of *Cymodocea serrulata, Halophila ovalis* and *Halodule uninervis* were collected using SCUBA from depths of between 2 and 5 m. Plants were kept in insulated boxes under constant aeration and transported back to aquaculture facilities at The University of Western Australia in Perth, 850 km south of Shark Bay.

### Experimental Design

Seagrasses were pruned to five shoots and all roots removed to ensure new root growth and to avoid necrosis before being planted into pots submerged in 1,800 L tanks. Each tank operated as a separate recirculating system with filtered (25 μm) aerated seawater (∼35 psu) and was held at a constant temperature of 26°C. Three plants of each species were planted into pots to emulate a mixed meadow, with each pot treated as a single replicate for each species (i.e., one replicate pot contained three separate ramets of each species). Siliceous river sand mixed with 1.5% dry weight of beach wrack (dried and ground seagrass leaves) was used as sediment to emulate natural sediment conditions for microbial colonization, as well as to benefit the growth of seagrasses (Statton et al., 2013). The physical and chemical properties of this sediment are presented in Supplementary Table S1.

Plants were acclimated to tank conditions for 2 months (May-June; start of austral winter) prior to commencement of the experiment. The experiment was a factorial design consisting of three species grown at four light treatments with three replicate pots for microbiome sampling and three replicate pots for exudate collection per treatment. The light treatments were: continuous full light ∼8 moles photons m^-2^ day^-1^ (Control), continuous medium light ∼3 moles photons m^-2^ day^-1^ (Medium), continuous low light ∼1 moles photons m^-2^ day^-1^ (Low), and fluctuating light of 10 days at low light followed by 4 days at high light (Fluctuating). Light treatments were imposed for a total of 2 weeks using ambient light and shade cloth that covered the tanks. Light levels were monitored throughout the experiment using photosynthetically active radiation (PAR) cosine collectors (Onset Hobo PAR sensors attached to a HOBO micro-station logger). Average daily PAR and total PAR received in the four light treatments are presented in Supplementary Table S2.

### Root Exudate Collection and Characterisation

At the end of the 2 weeks of light treatments, three replicates of each seagrass species and treatment combination were removed from the sediment and the roots gently washed using a filter sterile artificial seawater solution (∼35 psu; Supplementary Table S3 for salt composition). The roots were then threaded through small holes in a polyethylene barrier to separate them from rhizome and shoot tissue and placed in 50 mL of the artificial seawater solution for 20 min. The entire trap solution was filtered through a 0.2 μm syringe filter and stored at 4°C until analyzed within 1 week. A blank solution of artificial seawater with no roots was also collected at this time and analyzed in the same manner. Root length was estimated using images captured with a Canon S110 that were then analyzed using WinRhizo software (version 4.1, Regent Instruments Inc., Quebec City, QC, Canada). Plants were then separated into shoots and roots, dried at 60° C for 72 h and weighed.

Root exudates were analyzed for dissolved organic carbon (DOC), total dissolved nitrogen (TDN), and dissolved organic matter (DOM) fluorescence excitation-emission matrix spectroscopy. Concentrations of DOC and TDN were determined by high temperature catalytic oxidation on a Shimadzu TOC-V total carbon and total nitrogen analyser (Shimadzu, Columbia, MD, United States). Samples were first acidified with HCl and concentrations were determined using an eight point calibration curve with a DOC and TDN standard. A blank and standard check was performed every 20 samples. All DOC and TDN concentrations are the mean of three-five replicate injections, with a variance of < 3%. Fluorescence excitation-emission matrix (EEM) spectroscopy in combination with parallel factor analysis (PARAFAC) was used to determine chemical characteristics of the DOM in the seagrass root exudates. PARAFAC-EEM allows the identification of particular fluorophores in a mixture (e.g., humic/fulvic compounds, tyrosine and tryptophan) based on peaks in fluorescence intensity (Murphy et al., 2013). Detailed procedures for PARAFAC analysis can be found in Stedmon and Bro (2008). Briefly, samples were diluted to correct for inner filter effects (Green and Blough, 1994) and fluorescence intensity measured across emission wavelengths ranging from 300 to 600 nm (2 nm increments) and excitation wavelengths ranging from 240 to 540 nm (5 nm increments). Excitation and emission slit widths were 5 nm and the photomultiplier tube voltage was set to 725 V. All EEMs were blank subtracted and Ramen normalized using the area under the water Ramen peak at the excitation wavelength of 350 nm collected from the blank artificial seawater solution. EEMs were also corrected for instrument bias using files provided by Varian, and normalized by their maximum fluorescence values prior to PARAFAC modeling to reduce the influence of highly concentrated samples. PARAFAC was used to model major components of EEMs using the DOMFluor toolbox (version 1.7) and the N-way Toolbox (version 3.1) in MATLAB (version 8.5.0.197613). Fluorescence EEMs were measured and recorded using a Varian Cary Eclipse fluorometer (Varian Inc., Mulgrave, VIC, Australia).

### DNA Collection, Extraction and 16S Illumina Sequencing

An additional three pots per treatment were harvested at the end of the 2 weeks for microbiome sampling. Prior to harvesting plants, sediment cores were taken adjacent to the apical shoot of each species using small 10 mL syringes with the top removed (i.d., ∼1 cm). The small size of the cores ensured no roots were accidently sampled. One core was sampled per plant and light treatment combination and frozen at -80°C. Then, plants were removed from sediments and unwashed roots arising from the apical shoot were cut from the rhizome, placed in Eppendorf tubes and frozen at -80°C. Any remaining soil particles were gently scraped off the root surface using a sterile spatula immediately prior to DNA extraction. Hence the root microbiome in this study is defined as endosphere + rhizoplane inhabiting microorganisms.

DNA was extracted from ∼0.5 g of wet sediment (2 × extractions and DNA pooled) and ∼0.1 g of root tissue using the MoBio PowerSoil DNA extraction kit according to manufacturer’s instructions. A Fast Prep 24 (MP Biomedicals) set at 6.5 m s^-1^ for 80 s was used to disrupt the soft seagrass roots and to lyse cells with DNA eluted in nuclease-free water. Microbial communities were profiled using the primers 341F – 806R that target the V3-V4 hypervariable region of the 16S rRNA gene and have lower off-target amplification of plant chloroplasts (Muyzer et al., 1993; Muhling et al., 2008; Caporaso et al., 2011). Sequencing of pooled amplicons was performed by the Australian Genome Research Facility (AGRF) on the Illumina MiSeq platform, using Nextera XT v2 indices and 300 bp paired end sequencing chemistry.

Bioinformatic analysis of sequence reads was largely completed following the pipeline developed by Comeau et al. (2017). Paired-end reads were assembled by aligning the forward and reverse reads by their common overlapping parts using PEAR (version 0.9.1) (Zhang et al., 2014). Quality metrics were checked using FastQC (version 0.11.5) and filtered using tools in FASTX (version 0.7) and BBmap (version 35.84). Ambiguous and chimeric sequences were identified and removed using VSEARCH (version 1.4.0) with the Ribosomal Database Project as reference (Rognes et al., 2016). All downstream analyses were performed in QIIME (version 1.9.1) (Caporaso et al., 2010b). Open-reference OTU picking was performed using the SortMeRNA (version 2.0) method (Kopylova et al., 2012) with a minimum identity of 97%. Taxonomy was assigned using UCLUST (version 1.2.22) (Edgar, 2010) with the greengenes database as reference (version 13.8) and sequences were aligned using PyNAST (version 1.2.2) (Caporaso et al., 2010a). OTU’s identified in less than 0.1% of the reads were removed as well as reads identified as chloroplast or mitochondria. Archaea were not filtered out. PICRUSt (version 1.0.0) analysis was then used to predict metagenome functions of the microbial community after filtering *de novo* OTU’s from the open reference table and normalizing by 16S copy number (Langille et al., 2013). Metagenomes were predicted against the KEGG database and KEGG pathways created at level 2 and 3.

### Scanning Electron Microscopy (SEM)

An additional set of plants were collected from the sample sites in Shark Bay in November 2016 for examination of natural microbial colonization on roots using scanning electron microscopy (SEM). For these samples, plants were carefully removed from the sediment, then apical roots were removed and immediately placed in a solution of seawater-glutaraldehyde (2.5%) and left to fix overnight at room temperature. Fixed roots were then stored at 4°C until analysis on the SEM. For SEM examination, three replicate apical roots were cut into 0.5 cm sections comprising root tip, elongation zone and the mature root hair zone. Samples were then dehydrated in an ethanol series (50, 70, 95, and 100%) and by critical point drying with liquid CO_2_. Samples were then coated with Pt and a 2 nm layer of C and imaged on a Zeiss 1555 VP-FESEM microscope with the secondary electron signal of 5 Kv. Twenty random fields of view were imaged for each root region.

### Data Analysis

Differences in the predicted (PICRUSt) metagenome functions between roots and sediments and among plant species were analyzed with two-sided Welch’s *t*-test and Benjamini–Hochberg correction for multiple testing using the Statistical Analysis of Metagenomic Profiles (STAMP; version 2.1.3) (Parks and Beiko, 2010). All other statistical analysis was carried out in R studio (version 0.99.902) using the Phyloseq (version 1.14.0) (McMurdie and Holmes, 2013), ggplot2 (version 2.2.1) (Wickham, 2009) and vegan packages (version 2.4-3) (Oksanen et al., 2016). Bacterial diversity within samples (alpha diversity) was estimated using Shannon and Chao 1 diversity indices. Weighted Unifrac of relative abundances were used to construct dissimilarity matrices of the communities (beta-diversity) and visualized using PCoA. Stratified permutational multivariate analysis of variance (PERMANOVA; R vegan function adonis) with 999 permutations was conducted to explore the percentage of variance that could be explained by the differences in beta diversity among plant species and sample type (sediment versus root). Differences in alpha diversity indices among plant species and sample type (fixed factors) were compared using linear mixed effects models (tank position as random effect) using the nlme package (version 3.1) with Johnson transformed data (Johnson version 1.4) to conform to parametric assumptions. Differences in root exudates (PARAFAC-EEM components, DOC or TDN) and biomass among plant species and by light treatment were also analyzed using linear mixed effects using log transformed data where required.

To test the hypothesis that light impacts root microbial communities, root samples were analyzed with canonical correspondence analysis (CCA) (constrained by plant species and light). Monte Carlo permutation tests (with 999 permutations) were used to test the significance of the variation in species composition explained by the variables of the CCA.

Differential abundance of OTUs between plant roots and sediments, among plant species and between control light and low light treatments within the root samples was performed on variance stabilized data that was agglomerated to either family or genus level using the DESeq2 package (version 1.10.1) (McMurdie and Holmes, 2014). Significance was determined by Benjamini–Hochberg corrected *P*-values < 0.01. All raw sequences have been uploaded to NCBI Sequence Read Archive (SRA) under submission number SUB2987535.

## Results

### Microbial Diversity, Composition and Predicted Function of Seagrass Roots

**FIGURE 1:**
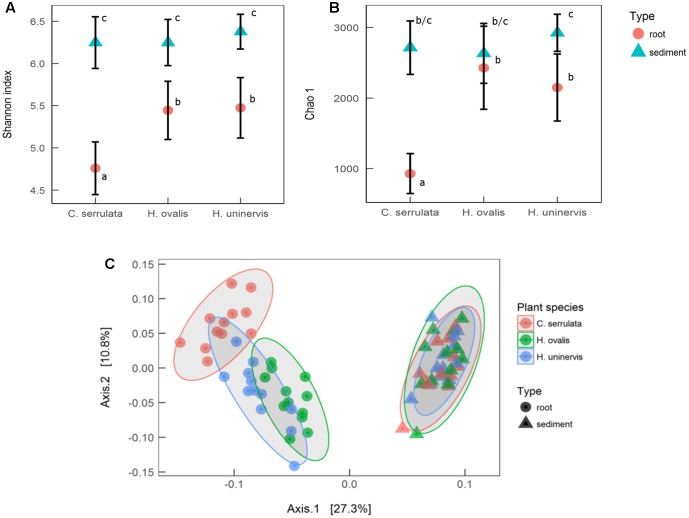
Alpha and beta diversity of microbial communities associated with seagrass roots and surrounding sediment. **(A)** Mean species richness (Shannon index) ± 1 SD, *n* = 12. **(B)** Mean estimated abundance (Chao 1) ± 1 SD, *n* = 12. Significant *post hoc* comparisons are indicated by lowercase letters. **(C)** Principal coordinates analysis (PCoA) of weighted UniFrac distances with 95% confidence ellipses around each seagrass species in roots and sediments.

Seagrass root microbiomes exhibited lower alpha diversity (Shannon index) than those in surrounding sediments (*F*_1,66_ = 137, *p* = 0.001, **Figure 1A**). With the exception of *H. ovalis*, the estimated total abundance of the microbial community (Chao 1) was also reduced in seagrass roots compared to surrounding sediments (*F*_1,66_ = 53, *p* < 0.001, **Figure 1B**). Of the three seagrass species, microbial communities associated with *C. serrulata* roots had the lowest species diversity and estimated abundance. In contrast, there was no difference in the sediment only samples for either measures of alpha diversity (Shannon or Chao 1) (**Figures 1A,B**).

PCoA of weighted UniFrac distances revealed that the composition of the root microbiome was strongly affected by the presence of seagrass roots (PERMANOVA; *F*_1,66_ = 27.3, *p* < 0.001). Root microbiomes were also differentiated by plant species (PERMANOVA; *F*_2,66_ = 3.2, *p* = 0.001), with *H. ovalis* and *H. uninervis* appearing more similar than *C. serrulata* (**Figure 1C**). In contrast, there was no differentiation of microbial communities in the samples of sediments without roots (**Figure 1C**).

**FIGURE 2:**
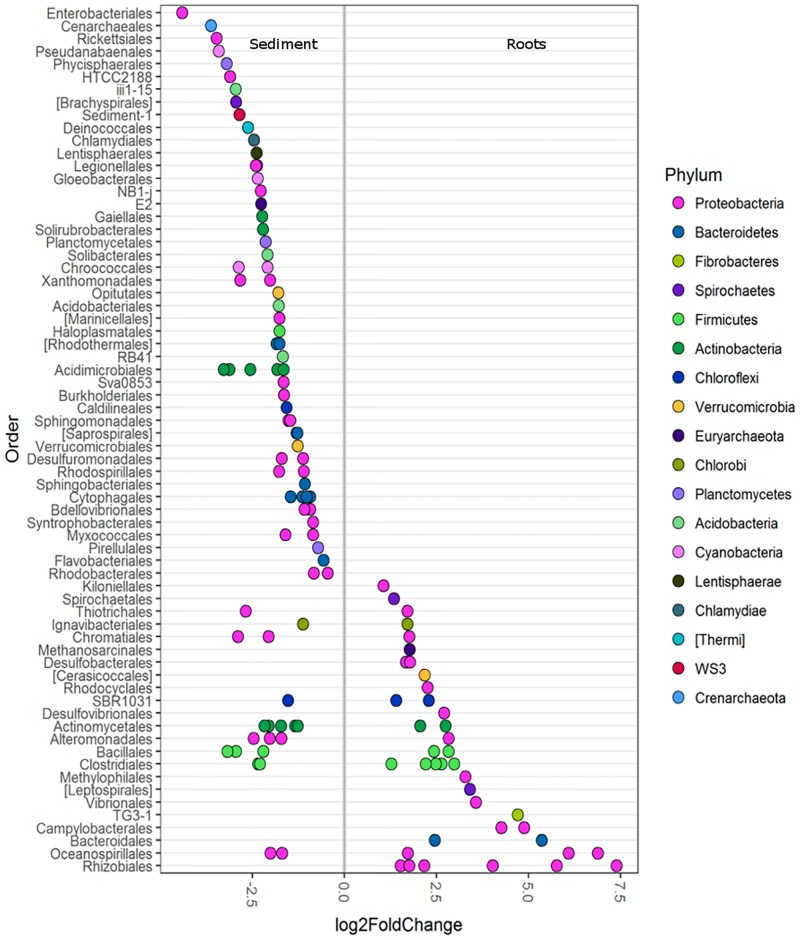
Differentially abundant OTUs (agglomerated by family level) between seagrass roots (species combined) and sediment displayed at the order level. Only OTUs with a significance of *p* < 0.01 are shown. A greater than zero log2-fold-change indicates phyla that were more differentially abundant in roots. Each circle represents one family, not a single OTU. Multiple circles within an order indicate multiple families that were enriched.

Differential abundance analysis was used to investigate the main OTUs driving the differences in roots and sediments (with all seagrass species combined). Root microbiomes were enriched in the various classes of *Proteobacteria* (α, β, γ, ε, and δ) and *Bacteroidia* (**Figure 2**). Root samples were particularly enriched in a diversity of *Rhizobiales* (**Figure 2**). *Bacteroidales, Campylobacterales, TG3-1, Vibrionales, Leptospirales, Methylophilales, Desulfovibrionales, Rhodocyclales, Cerasicoccales, Desulfobacterales*, and *Kiloniellales* were also enriched in root samples (**Figure 2**). Conversely, sediment samples were more abundant in *Enterobacteriales, Rickettsiales, Planctomycetes, HTCC2188, Brachyspirales, Deinococcales, Lentisphaerales*, and *Cenarchaeales* Archaea (**Figure 2**).

**FIGURE 3:**
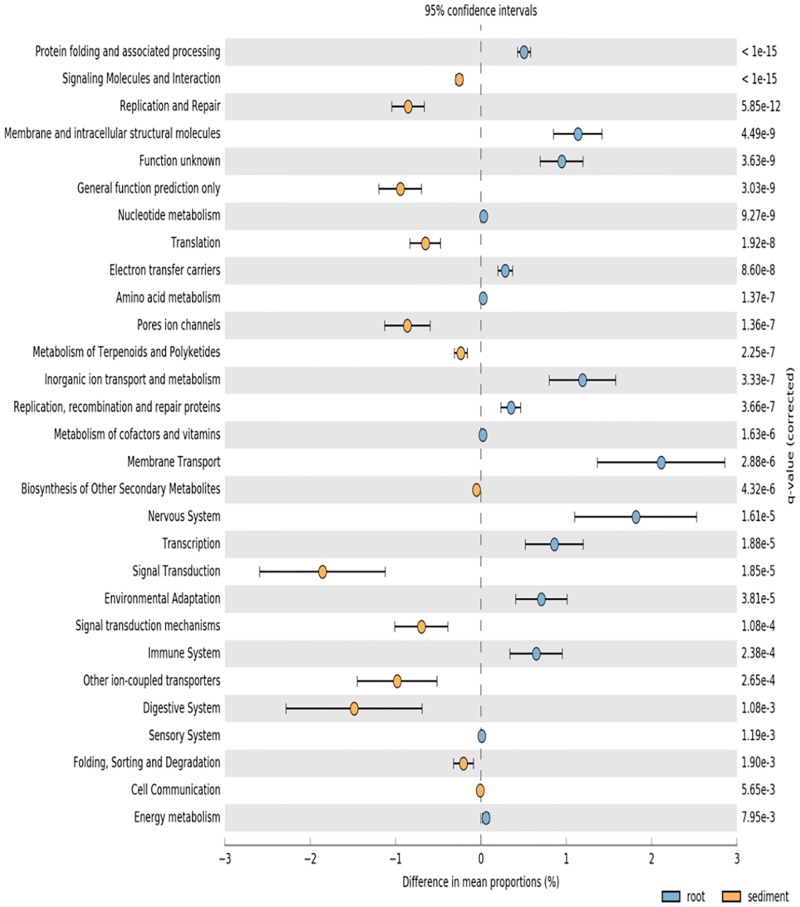
PICRUSt predicted KEGG pathways (level 2) of seagrass root associated microbial communities (blue) and sediment associated microbial communities (orange) at the significance level of *p* < 0.01.

PICRUSt analysis was used to predict potential function of root associated and sediment associated microbial communities based on the metagenome data of closely related taxa. Root microbiomes showed higher mean proportions of KEGG pathways associated with membrane transport and metabolism (particularly amino acids) (**Figure 3**). Conversely, sediment associated communities had higher proportions of KEGG pathways associated with signaling and cell communication as well as metabolism of terpenoids and polyketides (**Figure 3**).

**FIGURE 4:**
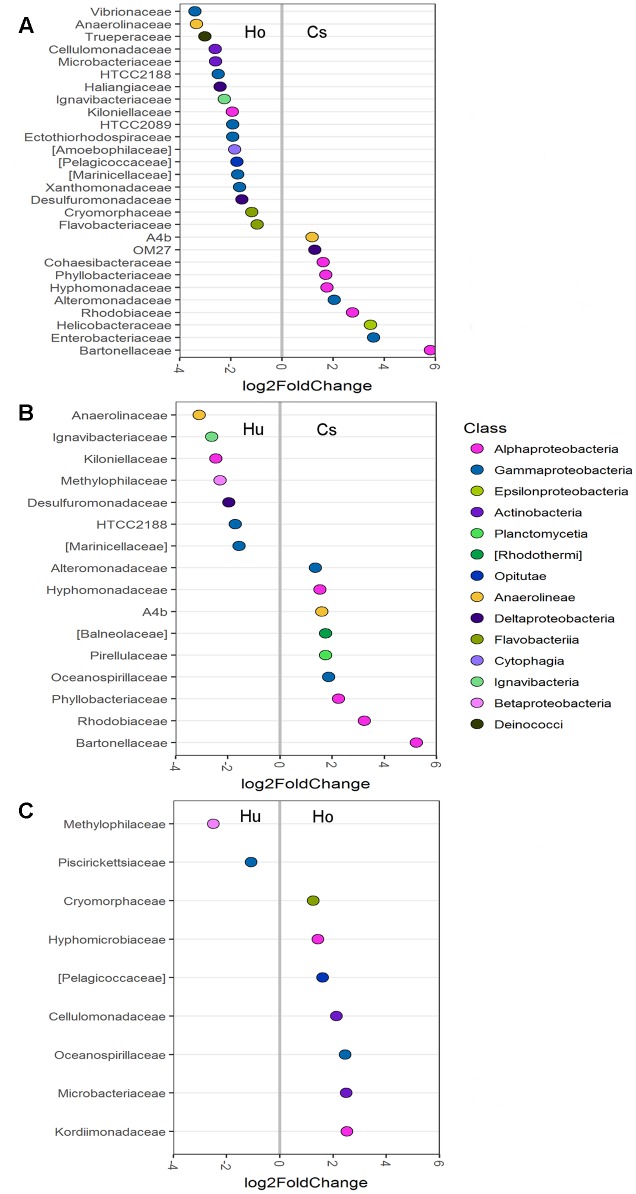
Differentially abundant OTUs (agglomerated by family) between root microbiomes of three seagrass species at the significance level of *p* < 0.01. **(A)** Comparison between *H. ovalis* (Ho) and *C. serrulata* (Cs), **(B)** comparison between *H. uninervis* (Hu) and *C. serrulata* (Cs) and **(C)** comparison between *H. uninervis* and *H. ovalis.*

Differential abundance analysis was also used to compare abundances in individual OTUs between the root microbiomes of each seagrass species (**Figure 4**). Root microbiomes of *C. serrulata* had a greater abundance of *Alphaproteobacteria* (including *Bartonellaceae, Rhodobiaceae*, *Hyphomonadaceae*, and *Phyllobacteriaceae*), *Alteromonadaceae* and *Anaerolineae (A4b)* compared to both *H. ovalis* and *H. uninervis*. Conversely, root microbiomes of both *H. ovalis* and *H. uninervis* had a greater abundance of *Anaerolinaceae*, *Ignavibacteriaceae*, *Kiloniellaceae*, *Desulfuromonadaceae*, *HTCC2188*, and *Marinicelleceae* compared to *C. serrulata. Cellulomonadaceae* and *Microbacteriaceae* within the *Actinobacteria* as well *Pelagicoccaceae* and *Cryomorphaceae* were more abundant in *Halophila ovalis* roots, whereas *Methylophilaceae* were more abundant in the roots of *H. uninervis* (**Figure 4**).

Differences in predicted functions among the root microbiomes of the three seagrass species reflected the patterns seen in alpha and beta diversity, whereby predicted functions of *H. ovalis* and *H. uninervis* were more similar than that of *C. serrulata* (Supplementary Figure S1). Microbial communities associated with the roots of *H. ovalis* and *H. uninervis* showed higher mean proportions of KEGG pathways associated with of cell motility, metabolism and signaling compared to *C. serrulata* (Supplementary Figure S1). Conversely, those communities associated with *C. serrulata* roots had higher proportions of KEGG pathways associated with cell growth and death, biodegradation of xenobiotics and secondary metabolites and amino acid metabolism (Supplementary Figure S1). There were few differences in predicted functions between *H. ovalis* and *H. uninervis* (Supplementary Figure S1).

**FIGURE 5:**
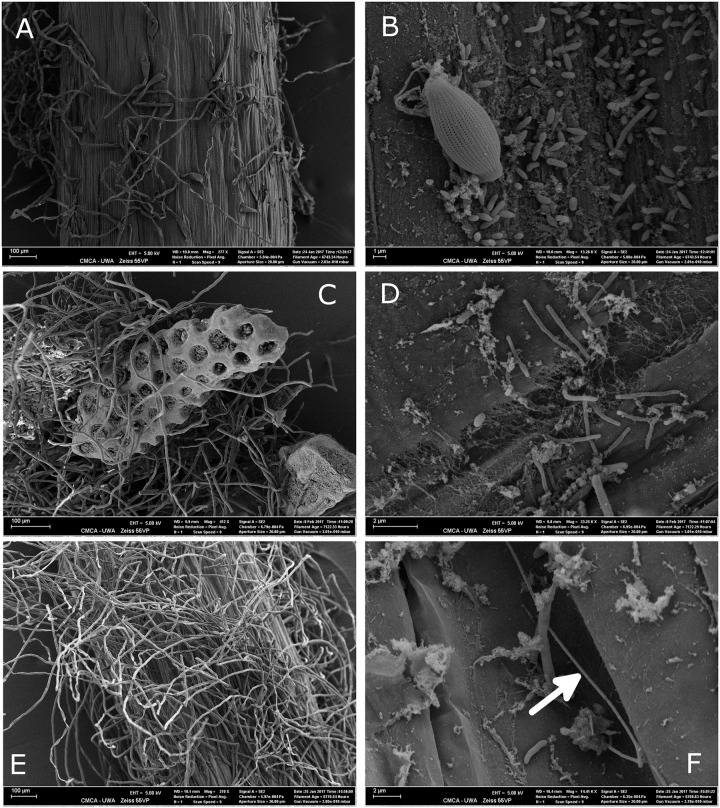
Scanning electron micrograph (SEM) of the root hair zone of three seagrass species, *Halophila ovalis, Halodule uninervis*, and *Cymodocea serrulata* collected from Shark Bay. **(A)** Root topology of the root hair zone of *C. serrulata*. **(B)** Microbial cells and a diatom attached to the surface of *C. serrulata.* **(C)** Root hairs of *H. uninervis* growing through biogenic sediment particles. **(D)** Microbial colonization of *H. uninervis* showing a diversity in cell types (coccus, bacillus and filamentous). **(E)** Root topology of the root hair zone of *H. ovalis* showing numerous long root hairs. **(F)** Microbial colonization of *H. ovalis* showing a long filamentous bacteria (arrow). These filaments were absent from *C. serrulata*.

Microbial colonization, as examined by SEM, showed a patchy distribution with the largest observed density of microbes occurring in the mature root hair zone (**Figure 5** and Supplementary Figure S2). The elongation zone of all three species was mostly devoid of microbial cells (Supplementary Figure S3). Filamentous bacteria and a diversity in cell types were observed on roots of *H. ovalis* and *H. uninervis* (**Figure 5** and Supplementary Figure S2). Additionally, the root hairs of both *H. ovalis* and *H. uninervis* were longer and more numerous than *C. serrulata* and often grew through sediment particles binding them closely to the root (**Figure 5**). Diatoms were only observed attached to *C. serrulata* roots (**Figure 5** and Supplementary Figures S2, S3).

### Seagrass Root Growth and Exudation under Reduced Light

**FIGURE 6:**
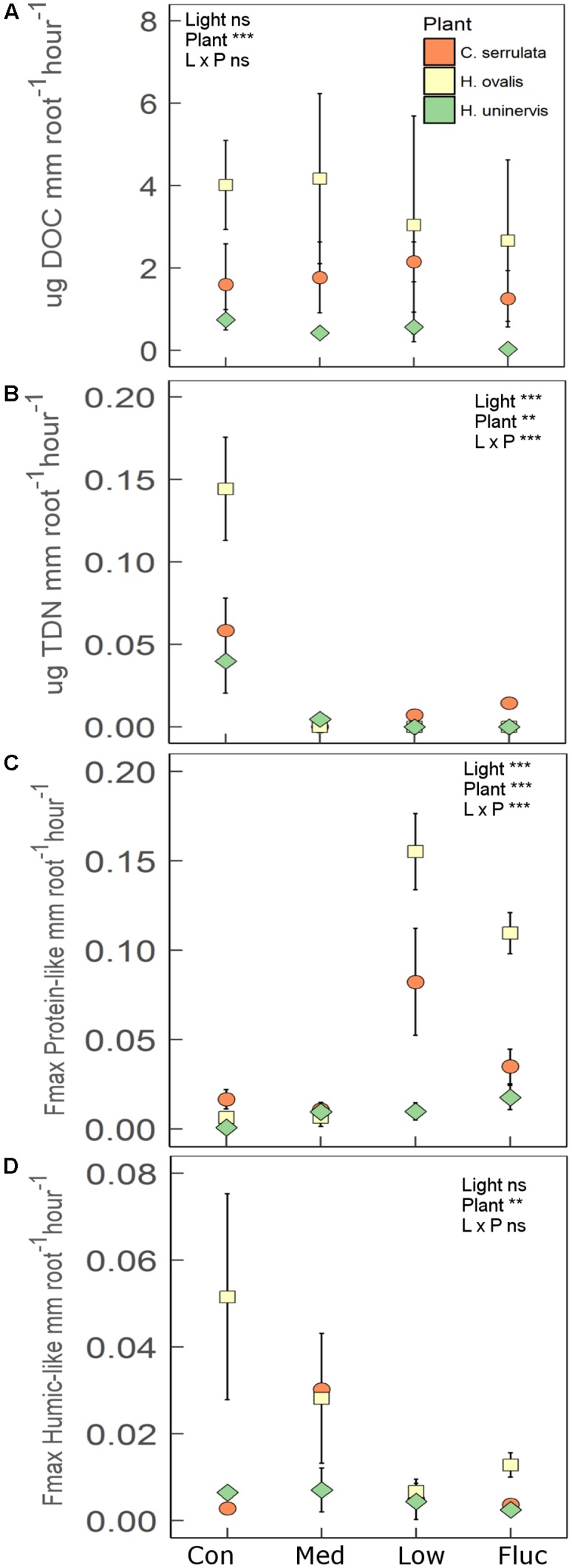
Root exudation for three species of seagrass grown for 2 weeks under four light treatments, control (Con), medium (Med), low (L), and fluctuating (Fluc). **(A)** Dissolved organic carbon (DOC; *n* = 3), **(B)** total dissolved nitrogen (TDN; *n* = 3), **(C)** protein-like components (*n* = 3), and **(D)** humic-like components (*n* = 3). Values are means ± 1 SD. The main effect of light treatment, plant species and interaction is indicated by the ^∗^ in each panel, where ^∗^*p* < 0.05, ^∗∗^*p* < 0.01, ^∗∗∗^*p* < 0.001, ns = not significant.

Root length and root biomass of all three seagrass species were unaffected by any of the light treatments following the 2 weeks experimental period (Supplementary Table S4). There was also no significant changes in aboveground growth (shoot biomass, leaf length and width) in response to light treatments. Additionally, photosynthetic performance of all seagrass species (assessed using rapid light curves) was also largely unaffected by light treatments (Supplementary Table S6). In contrast, the rate of root exuded TDN was significantly lower in reduced light for all species compared to the control (**Figure 6B**). This effect was particularly pronounced for *H. ovalis*, where TDN exudation was reduced by 15-fold (**Figure 6B**). A total of five fluorescent components were validated from the PARAFAC model (Supplementary Figure S4). These components were classified as protein-like (C1 and C3), humic-like (C2 and C4) or left unclassified (C5) based on comparisons between peak emission and peak excitation from this study to those previously reported (Supplementary Table S5). All seagrass species significantly increased the rate of root exuded protein-like DOM when grown under constant low light and fluctuating light as compared to the control (**Figure 6C**). In contrast, the rate of root exuded DOC and humic-like DOM was not affected by light treatment for any of the seagrass species (**Figures 6A,D**). However, there were significant differences in the rate of root exuded DOC and humic-like DOM among seagrass species, with *H. ovalis* exudation of DOC being two-fold higher than that of *C. serrulata* and seven-fold fold higher than that of *H. uninervis* (**Figure 6B**). *Halophila ovalis* also exuded a greater rate of TDN and protein-like DOM than did *C. serrulata* and *H. uninervis* and a greater rate of humic-like DOM than *H. uninervis* (**Figure 6**). Root exudation of the unclassified component (C5) was also not affected by light treatment (*F*_3,20_ = 1.3, *p* = 0.29), but was exuded at a significantly greater rate from *C. serrulata* roots than from roots of either *H. ovalis* or *H. uninervis* (Supplementary Figure S5).

### Effect of Light Treatment on Seagrass Root Microbiomes

**FIGURE 7:**
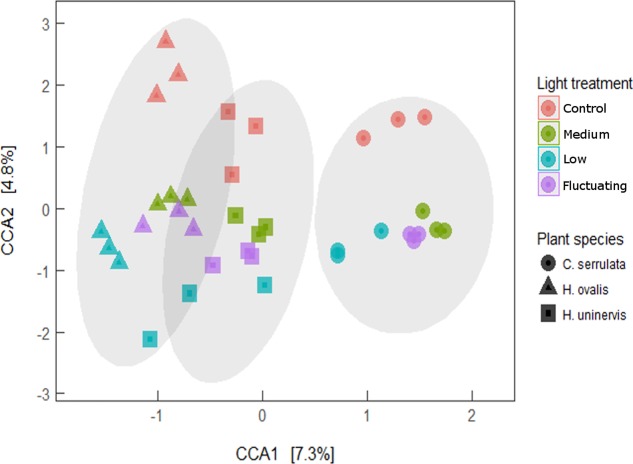
Canonical correspondence analysis (CCA) of seagrass root microbiomes under four light treatments with 95% confidence ellipse around each seagrass species.

Canonical correspondence analysis (CCA) revealed that the entire composition of the root microbiome of each seagrass species grown under any of the light treatments was distinct from that of those grown under full light (**Figure 7**). Although the variation in community composition explained by light and plant species of the CCA model was relatively small (4.8 and 7.2%, respectively), the model was still found to be significant (Monte Carlo permutation; *F*_5,30_ = 1.78, *p* = 0.001). The lowest light treatment tended to group furthest away from the control for all seagrass species, although this was more apparent for *H. ovalis* and *H. uninervis* than for *C. serrulata* (**Figure 7**). Root microbiomes still clustered by seagrass species as in the unconstrained PCoA plot (**Figure 1**), that is, *H. ovalis* and *H. uninervis* grouped more closely than *C. serrulata* (**Figure 7**).

**FIGURE 8:**
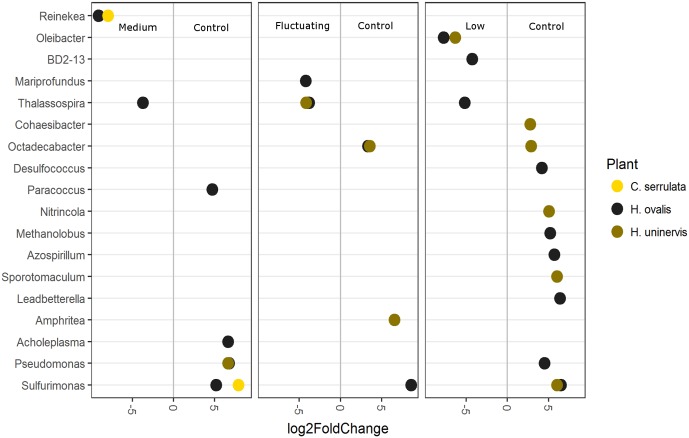
Differentially abundant OTUs (agglomerated by genus; significance of *p* < 0.05) between the full light control and each light treatment. Greater than 0 log2-fold-change indicates OTUs more differentially abundant in the control.

To further explore which OTUs may be driving the responses to light reduction observed in the CCA plot, differential abundance was used to compare each light treatment with the full light control (**Figure 8**). As in the CCA plot, constant low light had the largest number of OTUs that were differentially abundant compared to the control, but this was only the case for *H. uninervis* and, in particular, for *H. ovalis* (**Figure 8**). Conversely, *C. serrulata* only had differences in OTUs when comparing the medium light treatment to the control (**Figure 8**). Although there were differences in the response of individual OTUs among each seagrass species, some general trends can be described. Bacteria considered to be aerobic, or micro-aerobic were more abundant in the full light controls including *Pseudomonas, Amphrita*, *Azospirillum, Leadbetterella*, *Cohaesibacter, Nitrincola* – although these trends were not necessarily consistent between the seagrass species (**Figure 8**). *Sulfurimonas* was especially more abundant in the control light as compared to all of the reduced light treatments (**Figure 8**). Despite the differences in OTUs, PICRUSt analysis revealed no corresponding change in predicted metagenomes of root microbiomes between any reduced light treatments and full light.

## Discussion

This is the first study to examine root microbiomes of seagrasses and their interaction with aboveground light reduction, even though lower light availability is recognized as a contributing factor to their worldwide decline (Waycott et al., 2009). We found that light reduction altered seagrass root exudation and the composition of seagrass root microbiomes with a decrease in abundance of potentially beneficial microorganisms. These changes occurred after only 2 weeks of light treatment and before any changes in root growth, implying that root exudation and alterations in root microbiomes respond rapidly to declines in light availability and could be used as an early indicator of light stress.

### Microbial Diversity, Composition and Function Is Strongly Influenced by the Presence of Seagrass Roots

As hypothesized, microbial composition and predicted function differed among seagrass species, as well as between seagrass roots and the surrounding sediment. Seagrasses, like their terrestrial counterparts, exert selective pressures on their root microbiomes that result in communities that are distinct not only from the sediment but also among individual plant species; even when growing as a mixed meadow. The microbial phyla that were more abundant in the roots of the seagrasses studied here (such as *Proteobacteria* and *Bacteroidetes*) are phyla that also dominate root microbiomes of terrestrial plants (Chen et al., 2016; Wagner et al., 2016; Hartman et al., 2017; Wemheuer et al., 2017). Seagrass roots were also markedly enriched in *Rhizobiales*, a taxonomic order that includes a diversity of nitrogen-fixing microbes that form symbiotic relationships with terrestrial plants (Krol et al., 2011; Fischer et al., 2012). While these microbes are often assumed as being selected for as they aid plant survival [for example in the provision of nitrogen (Bouffaud et al., 2016)], the enrichment of these microbial groups could reflect historical evolution (i.e., from before seagrasses returned to the sea), or simply be a reflection of the ubiquity of those groups that are able to respond to and thrive within the strong concentration gradients typical of a rhizosphere environment.

Despite the similarities between the root microbiomes of seagrasses and terrestrial plants, there are some fundamental differences, particularly in regards to the enrichment of *Deltaproteobacteria* in seagrass roots. *Deltaproteobacteria* include many bacteria that are known sulfate reducers. Various groups of sulfate reducing bacteria have been isolated from multiple seagrass species in both culture dependant (Küsel et al., 1999, 2006; Cifuentes et al., 2003; Smith et al., 2004) and independent studies (Cúcio et al., 2016; Garcias-Bonet et al., 2016; Ettinger et al., 2017; Fahimipour et al., 2017; Trevathan-Tackett et al., 2017) and their enrichment on seagrass roots points to the predominately anoxic, sulfide-rich sediments that seagrasses colonize. Despite the potential for reduced sulfide accumulation to be toxic to seagrass metabolism (García et al., 2013), this functional group of bacteria is important in carbon mineralisation and ultimately in nutrient cycling in seagrass sediments (Holmer et al., 2001). Additionally, there is evidence to suggest that numerous sulfate reducing bacteria are also capable of nitrogen fixation (Bagwell et al., 2002; Devereux, 2005; Garcias-Bonet et al., 2016). Another group that was enriched in seagrass roots relative to the sediment was the *Campylobacterales. Campylobacterales*, and more generally, *Epsilonproteobacteria* (to which *Campylobacterales* belong), have previously been found enriched in the roots of the seagrasses *Zostera* spp. (Jensen et al., 2007; Cúcio et al., 2016; Ettinger et al., 2017) and *Halophila stipulacea* (Mejia et al., 2016), in the roots of the salt marsh plant *Spartina alterniflora* (Thomas et al., 2014), and in mangrove roots and sediments (Gomes et al., 2010; Andreote et al., 2012). *Campylobacterales* isolated from *Spartina* roots were identified as nitrogen fixers (McClung and Patriquin, 1980) and so both sulfate reducing bacteria and *Campylobacterales* may be important bacterial groups in the provision of nitrogen to marine and brackish water inhabiting plants.

Seagrass root microbiomes exhibited lower alpha diversity (Shannon and Chao 1) compared to surrounding sediments. The decreased diversity of root microbiomes compared to surrounding soil is a pattern typically exhibited for terrestrial plants (Bulgarelli et al., 2012; Chen et al., 2016; Rascovan et al., 2016; Hartman et al., 2017), but has also been confirmed for seagrasses and mangroves (Aravindraja et al., 2013; Ettinger et al., 2017) and again points to the selective pressures exerted by plant roots. Despite decreased microbial diversity, microbial metabolic activity in seagrass rhizospheres is typically higher than in bare sediment, presumably due to the provision of root exudates and other cellular debris (Karen et al., 1998; Devereux, 2005; Duarte et al., 2005). In this study, the higher proportions of KEGG pathways involved in membrane transport and metabolism (particularly amino acids) that were predicted from the seagrass root-associated communities, suggests a microbial population that is taking advantage of the higher availability of C and N sources that surround roots compared to the sediment.

Seagrass root microbiomes not only differed from those in sediments in their composition and predicted function, but also differed among the seagrass species, with each species harboring a unique root microbiome. This observation is in contrast with Cúcio et al. (2016) who found no difference in rhizosphere microbiomes among three seagrass species (*Zostera marina, Zostera noltii*, and *Cymodocea nodosa*) collected from the same location. This inconsistency can be largely explained by differences in sampling strategy where Cúcio et al. (2016) extracted DNA from soil attached to the roots (deemed rhizosphere), whereas in our study, DNA was extracted from the entire root after it had been scraped clean of sediment particles and hence the DNA includes that of rhizoplane and endosphere microbes. Analysis of whole-root-associated communities will be more sensitive to differentiation by host species due to the larger influence of the host on these closely associated communities compared to loosely bound rhizosphere soil. For this reason, careful evaluation of sampling strategy must always be considered in root microbiome research (Richter-Heitmann et al., 2016).

In this study, the influence of the seagrass host may be driven, in part, by the differences in exudation profiles. For example, *C. serrulata* harbored the most distinctive root microbiome; that is, it was most different in both composition and predicted function from the sediment and from *H. ovalis* and *H. uninervis*. One possible explanation is that the root exudates of *C. serrulata* contained the highest concentration of the component C5. C5 may be a type or mixture of tannin or phenolic-compounds (Krauss et al., 1994; Lattanzio, 2013). Secondary metabolites rich in phenolics and amino acids present in root exudates of the model plant *Arabidopsis* were previously found to have a greater influence on the rhizosphere microbiome than sugars (Chaparro et al., 2014). The high concentration of C5 may also explain why, in the current study, there was an increased proportion of KEGG pathways related to metabolism of xenobiotics and secondary metabolites in the *C. serrulata* root microbiome compared to *H. uninervis* and *H. ovalis*. Another explanation for the observed host specificity of the root microbiomes could lie in the differences in root topology among the three seagrass species. Both *H. uninervis* and *H. ovalis* have long numerous root hairs that bind sediment grains and, along with root and microbial-mucilage, contribute to the formation of what is typically defined as a rhizosheath in the terrestrial plant literature (Reinhold-Hurek et al., 2015; Jiayin et al., 2017). The lack of a tightly bound rhizosheath in *C. serrulata* may result in this species harboring more endophytic microbes relative to rhizoplane/rhizosheath inhabiting microbes, and may explain why *H. ovalis* and *H. uninervis* root microbiomes were more similar to the sediment both in terms of alpha and beta diversity. Consequently, the reduced structure of the rhizosphere in *C. serrulata* may also explain why the proportion of KEGG pathways associated with cell motility were lower in this species than either *H. ovalis* or *H. uninervis*, and why, filamentous bacteria, which are often highly mobile and capable of gliding (Dunker et al., 2011; Bjerg et al., 2016), were not observed on the surface of *C. serrulata* roots using SEM. However, as in all microbiome studies in which different host species or sample types are compared, there are potential contributing biases that are inherent in DNA extraction, PCR, sequencing and tissue fixation that can influence interpretation of results.

### Seagrasses Alter Root Exudation in Response to Reduced Light Availability

As hypothesized, seagrass root exudation was altered by a reduction in light available for photosynthesis, despite having no associated changes in root length or biomass. The root exudates of *H. ovalis* had the largest magnitude in response to reduced light availability, which is likely reflected in the greater sensitivity of this species to disturbance compared to *H. uninervis* or *C. serrulata* (Kilminster et al., 2015). Although there were species-specific patterns, in general, seagrasses decreased exudation of TDN, but increased exudation of protein-like components in both continuous low light and fluctuating light. Root exudation of DOC, protein-like and humic-like DOM was previously found to increase in these three seagrass species when grown under fluctuating light for 6 weeks (Martin et al., 2017). The increase in protein-like exudation could be due to release of fermentation products from the roots if they had become anoxic in low light, as oxygen supply is lowered with reduced photosynthetic activity (Smith et al., 1988; Greve et al., 2003). Additionally, in terrestrial plants, metabolic shifts under hypoxia and anoxia can impair ion transport across the root membrane, potentially making the roots more susceptible to leakage of exudates (Rittenhouse and Hale, 1971; Shabala et al., 2014). Lastly, as the roots were not sterile, some of the compounds were likely derived from the root microbiome itself. For example, the greater abundance of nitrogen fixing bacteria (e.g., *Azospirillum*) on roots of *H. ovalis* grown under 100% SI may have contributed to the greater TDN release from these roots in full light. Regardless, the response in the root exudation of seagrasses to light availability highlights one of the ways in which above-ground environmental disturbance can influence the below-ground environment.

### Composition of Seagrass Root Microbiomes Are Altered by Light Availability

As hypothesized, the composition of seagrass root microbiomes was altered by a reduction in light availability in a manner specific to each seagrass species. These species-specific changes could be related to the differences in the individual root exudation profiles of each seagrass species. For example, reduced light availability had the greatest effect on altering the root microbiome of *H. ovalis* (composition and individual OTUs), which was also the species that had the most significant change in exudation patterns when light availability was reduced. In contrast, we only saw a difference in genus-level OTUs of *C. serrulata* between the medium light treatment and the full light control– which also corresponded to the treatment that had the greatest change in exudation for this species. Whilst these results are only correlative, they suggest that changes in root exudation could be contributing to the changes in the microbiome and stress the potential importance of root exudates in mediating seagrass–microbe relations.

As for the majority of plants, the rhizosphere of seagrasses is a highly ephemeral and complex environment that is defined by a multiplicity of chemical gradients. However, one chemical gradient that is inherent to submerged rhizospheres is oxygen concentration. Seagrasses can lose oxygen from regions around their root tips creating a small oxic zone in an otherwise anoxic rhizosphere (Connell et al., 1999; Jensen et al., 2005). Lower light availability reduces the amount of oxygen that can be photosynthetically produced, subsequently decreasing the amount of oxygen that is ultimately leaked from the root tips into the rhizosphere (Brodersen et al., 2014; Jovanovic et al., 2015). This reduction in oxygen leakage could then affect microbial composition of the root microbiome by altering abundance and/or activity of aerobic versus anaerobic bacteria. Interestingly, many of the OTUs that were more abundant in the full light control compared to the reduced light treatments are considered aerobic or micro-aerobic (e.g., *Pseudomonas, Leadbetterella*, *Cohaesibacter, Nitrincola, Azospirillum*, and *Sulfurimonas*) (Steenhoudt and Vandereyden, 2000; Fallis, 2006; Gu and Mitchell, 2006; Abt et al., 2011; Turner et al., 2013; De-la-Peña and Loyola-Vargas, 2014; Joshi et al., 2016). This effect of reduced light availability on specific OTUs was particularly notable for *Sulfurimonas*, which are chemolithotrophs capable of sulfur oxidation (Campbell et al., 2006; Han and Perner, 2015). *Sulfurimonas* have previously been found enriched in the roots of *Zostera marina* collected over a large geographical range across the Northern Hemisphere (Fahimipour et al., 2017) and other from other *Zostera* spp. collected across Europe (Jensen et al., 2007; Cúcio et al., 2016), suggesting these putative sulfide oxidisers may prove to be wide-ranging keystone members of the seagrass root microbiome that are central to sulfide detoxification. Additionally, *Azospirillum* and some *Pseudomonas* are considered to have plant growth promoting properties due to their ability to either fix atmospheric nitrogen and/or produce growth promoting auxins (Steenhoudt and Vandereyden, 2000; Bagwell et al., 2002; Fallis, 2006; Gu and Mitchell, 2006). *Azospirillum* has previously been shown to exhibit directed movement toward oxygen concentrations (aerotaxis), which would be advantageous in enabling the bacteria to live in oxic niches that are optimal for nitrogen fixation (Barak et al., 1982; Zhulin et al., 1996). Whether the observed changes in OTUs with reduced light availability are a result of a possible reduction in oxygen leakage from roots in this study can only be speculated upon. However, the fact that putative plant growth promoting bacteria and sulfide oxidisers were less abundant in reduced light, means that the shift in the community could compound the negative effects of reduced light availability, particularly if light is reduced over prolonged periods. Further experimental efforts to uncover the effects of oxygen leakage on seagrass root microbial associated communities are now underway.

Despite changes in OTU abundance with reduced light, there was no corresponding change in predicted function. Functional redundancy in the community ensures key processes are unaffected by changes in community structure, which would be advantageous to seagrasses growing in environments experiencing frequent light deprivation. It is also possible that functional differences exist with reduced light, and that these differences could be driven by low abundant OTUs. Detection of functional differences within rare taxa might only be possible using deep sequencing or shotgun meta-transcriptomics. PICRUSt-predicted functional profiles can also be problematic for environmental microbiology owing to a lack of available reference genomes in the available databases. Lastly, as in metagenomics, the presence of a gene in community DNA does not denote that function is active; only transcriptomics and methods that specifically target capturing activity (e.g., SIP, NanoSIMS) can resolve true active functions. Regardless, PICRUSt-predicted functional profiles remain a useful tool for gaining functional insight in microbiome studies (Xu et al., 2014), and in the context of this study, suggests a level of functional redundancy in response to light disturbance.

## Conclusion

The main conclusions of this study in relation to our initial hypotheses are as follows: (1) the composition and predicted function of seagrass root microbiomes differ among species and are different to the surrounding sediment, (2) seagrass root exudation of TDN decreased, whilst protein-like DOM increased in low and fluctuating light availability, and (3) microbial composition, but not predicted function, is influenced by light availability in a seagrass-specific manner. Overall this study highlights the potential for above-ground light reduction to invoke a cascade of changes; from alterations in root exudation to a reduction in putative beneficial microorganisms and ultimately confirms the importance of the root environment – a critical, but often overlooked space.

## Author Contributions

BM: study design and conception, method development, data collection, data analysis, drafting the manuscript, and approval of final submission. DG: method development, drafting the manuscript and approval of final submission. JS: study design and conception, method development, data collection, drafting the manuscript, and approval of final submission. AS: method development, data analysis, drafting the manuscript, and approval of final submission. PG: method development, drafting the manuscript, and approval of final submission. MR: method development, drafting the manuscript, and approval of final submission. GK: study design and conception, method development, drafting the manuscript, and approval of final submission. All authors agree to be accountable for the content of the work.

## Conflict of Interest Statement

The authors declare that the research was conducted in the absence of any commercial or financial relationships that could be construed as a potential conflict of interest.
