# Supplementary material for: Low Light Availability Alters Root Exudation and Reduces Putative Beneficial Microorganisms in Seagrass Roots
Source: Front Microbiol. 2018 Jan 11;8:2667. doi: 10.3389/fmicb.2017.02667 (PMC5768916; doi:10.3389/fmicb.2017.02667)
Supplement: Supplementary file 1 [file Data_Sheet_1.DOCX]

Supplementary Material

Low light availability alters root exudation and reduces putative beneficial microorganisms in seagrass roots

Belinda C. Martin^1,2*^, Deirdre Gleeson^3^, John Statton^1,2,5^, Andre R. Siebers^1^, Pauline Grierson^1,4^, Megan H. Ryan^3^, Gary A. Kendrick^1,2,5^

^1^School of Biological Sciences, The University of Western Australia, 35 Stirling Highway, Crawley, WA 6009, Australia

^2^ The UWA Oceans Institute, The University of Western Australia, 35 Stirling Highway, Crawley, WA 6009, Australia

^3^ School of Agriculture and Environment, The University of Western Australia, 35 Stirling Highway, Crawley, WA 6009, Australia

^4^ West Australian Biogeochemistry Centre, School of Biological Sciences, The University of Western Australia, 35 Stirling Highway, Crawley, WA 6009, Australia

^5^ Western Australian Marine Science Institution, Perth, WA, Australia

*** Correspondence:**Belinda Martin
[Belinda.martin@research.uwa.edu.au](mailto:Belinda.martin@research.uwa.edu.au)

# Supplementary Figures and Tables

**Table S1.** Physical and chemical properties of the sediment

| Sediment property | Value |
| --- | --- |
| CaCO_3_ | 3.33 % |
| Porosity | 46.36 % |
| Bulk density | 1.52 g cm^-3^ |
| NH_4_-N | 1 mg kg^-1^ |
| NO_3_-N | < 1 mg kg^-1^ |
| Bicarbonate-extractable P | < 1 mg kg^-1^ |
| Bicarbonate-extractable K | 136 mg kg^-1^ |
| KCl-extractable S | 274 mg kg^-1^ |
| Organic C | 0.11 % |

**Table S2.** Average daily photosynthetically active radiation (PAR) and total PAR received in the four light treatments, full light control, medium light, low light and fluctuating light (10 days low, four days full light). Average daily PAR (moles photons m^-2^ day^-1^) is the average daily PAR (± 1 SD) and total PAR (moles of photons) is the sum of light received during the two week experimental period.

| PAR | Control | Medium | Low | Fluctuating |
| --- | --- | --- | --- | --- |
| Average daily PAR | 7.26 (2.30) | 2.95 (0.96) | 1.37 (0.84) | 3.14 (3.46) |
| Total PAR | 107 | 43 | 21 | 45 |

**Table S3.** Artificial seawater trap solution (~ 35 psu)

| Salt | g/kg |
| --- | --- |
| NaCl | 26.52 |
| MgCl_2_ | 2.45 |
| MgSO_4_ | 3.31 |
| CaCl_2_ | 1.14 |
| KCl | 0.73 |
| NaHCO_3_ | 0.20 |
| NaBr | 0.08 |


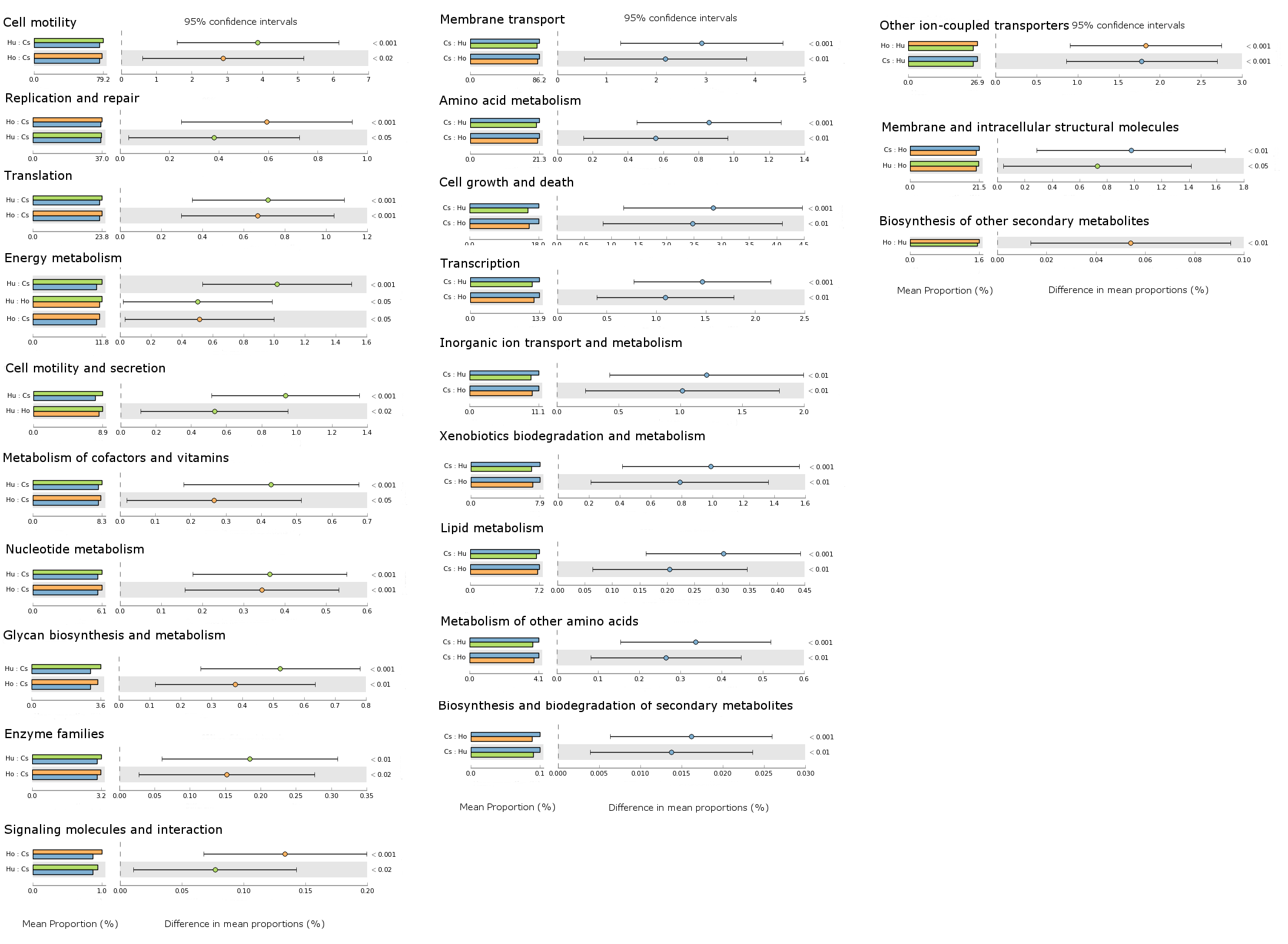


**Figure S1.** Comparison of PICRUSt predicted KEGG pathways (level 2) between root microbiomes of *Halophila ovalis* (orange), *Halodule uninervis* (green) and *Cymodocea serrulata* (blue).

**
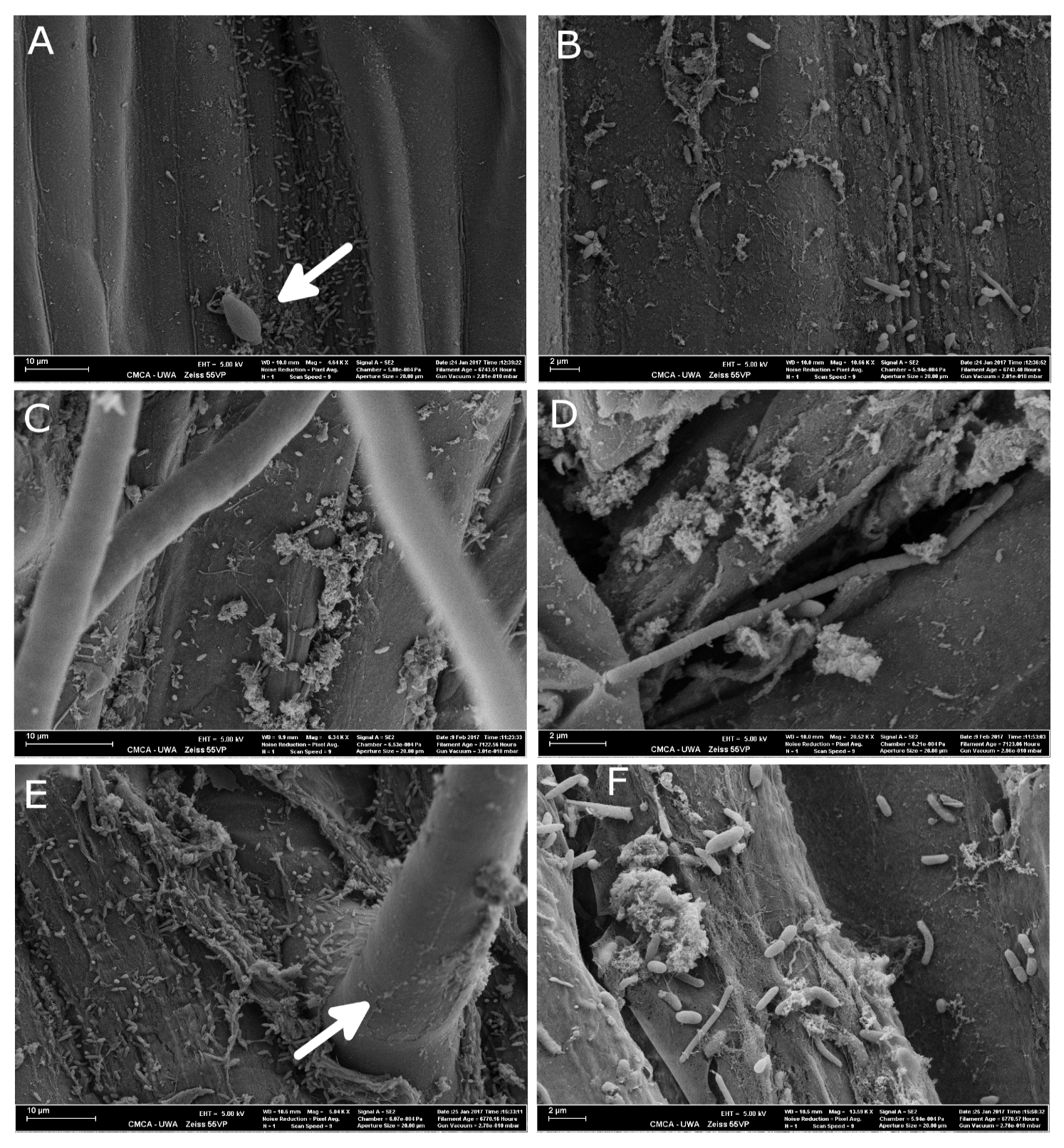
**

**Figure S2.** Scanning electron micrograph (SEM) of the root hair zone of three seagrass species, *Halophila ovalis, Halodule uninervis* and *Cymodocea serrulata* collected from Shark Bay. **(A)** Microbial colonization of *C. serrulata* root hair zone with diatom (arrow). **(B)** Microbial cells attached to the surface of *C. serrulata*. **(C)** Microbial colonization of *H. uninervis* roots with root hairs in foreground. **(D)** Filamentous bacteria on *H. uninervis* root. **(E)** Dense microbial colonization of *H. ovalis* with root hair (arrow). **(F)** Microbial colonization of *H. ovalis* showing a diversity of cell types.

**
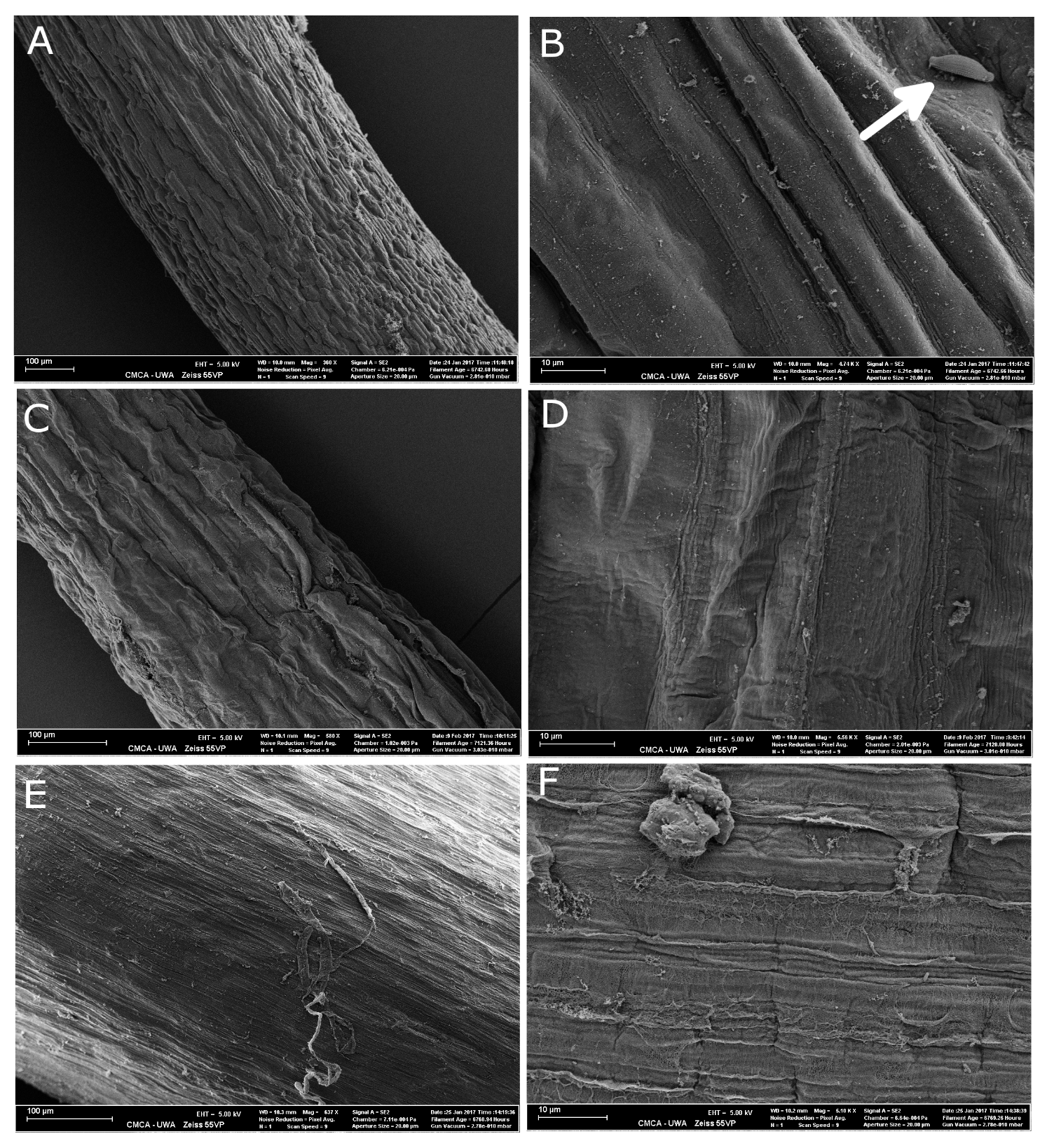
**

**Figure S3.** Scanning electron micrograph (SEM) of the elongation zone of three seagrass species, *Halophila ovalis, Halodule uninervis* and *Cymodocea serrulata* collected from Shark Bay. **(A)** Topology of the elongation zone of *C. serrulata*. **(B)** Sparse microbial colonization of the elongation zone of *C. serrulata*. A diatom is present (arrow). **(C)** Topology of the elongation zone of *H. uninervis* roots. **(D)** Sparse microbial colonization of the elongation zone of *H. uninervis*. **(E)** Topology of the elongation zone of *H. ovalis* roots. **(F)** Very few cells present in the elongation zone of *H. ovalis*.

**Table S4.** Root length and root biomass for three species of seagrass grown for two weeks under four light treatments, full light control, medium light, low light and fluctuating light (10 days low, four days full light). Values are mean (± 1 SD), *n* = 3).

| Species | Root parameter | Control | Medium | Low | Fluctuating |
| --- | --- | --- | --- | --- | --- |
| *Cymodocea serrulata* | Root length (mm) | 150 (54) | 118 (52) | 88 (102) | 166 (37) |
|  | Root DW (g) | 0.07 (0.03) | 0.05 (0.02) | 0.06 (0.04) | 0.07 (0.01) |
| *Halophila ovalis* | Root length (mm) | 43 (13) | 52 (31) | 27 (16) | 47 (5) |
|  | Root DW (g) | 0.02 (0.006) | 0.02 (0.009) | 0.01 (0.009 | 0.03 (0.001) |
| *Halodule uninervis* | Root length (mm) | 260 (53) | 198 (42) | 235 (72) | 291 (111) |
|  | Root DW (g) | 0.04 (0.007) | 0.03 (0.006) | 0.04 (0.01) | 0.04 (0.02) |


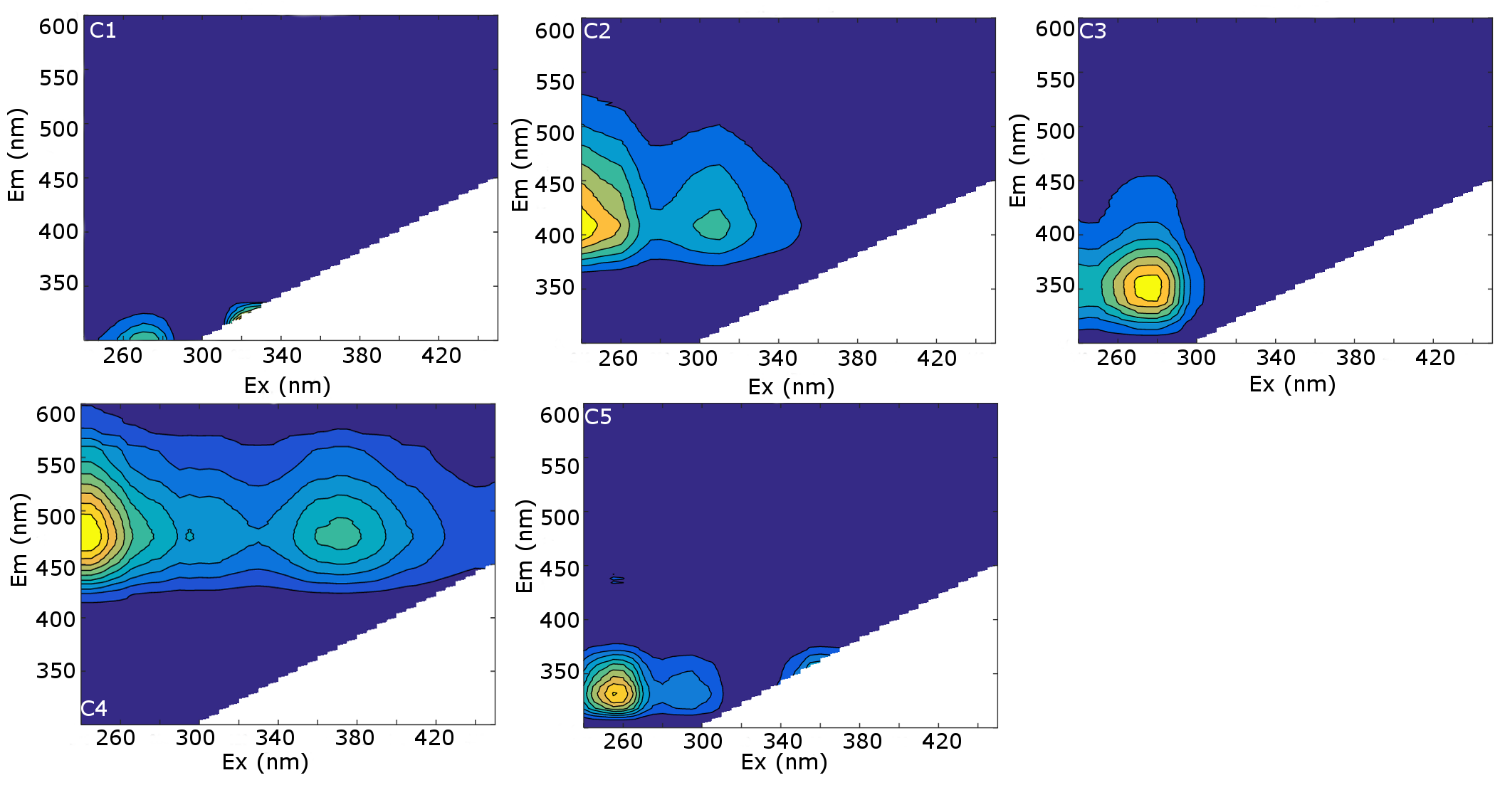


**Figure S4.** Excitation-emission spectra of PARAFAC components (C1 – C5) present in seagrass root exudates from *Cymodocea serrulata, Halodule uninervis* and *Halophila ovalis.*

**Table S5.** Classification of components validated from the PARAFAC model (C1 – C5) in this study based on published excitation/emission values for previously characterised components.

| Component | Peak excitation (nm) in this study | Peak emission (nm) in this study | Peak excitation (nm) | Peak emission (nm) | Classification |
| --- | --- | --- | --- | --- | --- |
| C1 | 265-275 | 300-312 | 270 | 300-305 | Tyrosine like^1,2,3,4^ |
| C2 | 240-250 (295-315)* | 395-425 (395-425) | < 250 (290-325) | 380-420 (425) | Humic-like, common in marine^1,2,3^ |
| C3 | 270-280 | 330-365 | 270-280 | 330-368 | Tryptophan-like^1,2,3^ |
| C4 | 240-250 (365-385) | 460-495 (460-490) | 237-260 (370) | 400-500 | Humic-like^1,2,3,4^ |
| C5 | 250-265 | 325-345 | 241 | 350 | Possibly Phenolic acids but largely unclassified^1,5^ |

***numbers in parenthesise are for a second smaller peak.**

1 **=** Jørgensen et al (2011)

2 **=** Fellman et al. (2010)

3 **=** Murphy et al. (2008)

4 **=** Yamashita et al. (2008)

5 **=** Airado-Rodŕiguez et al. (2009)

**
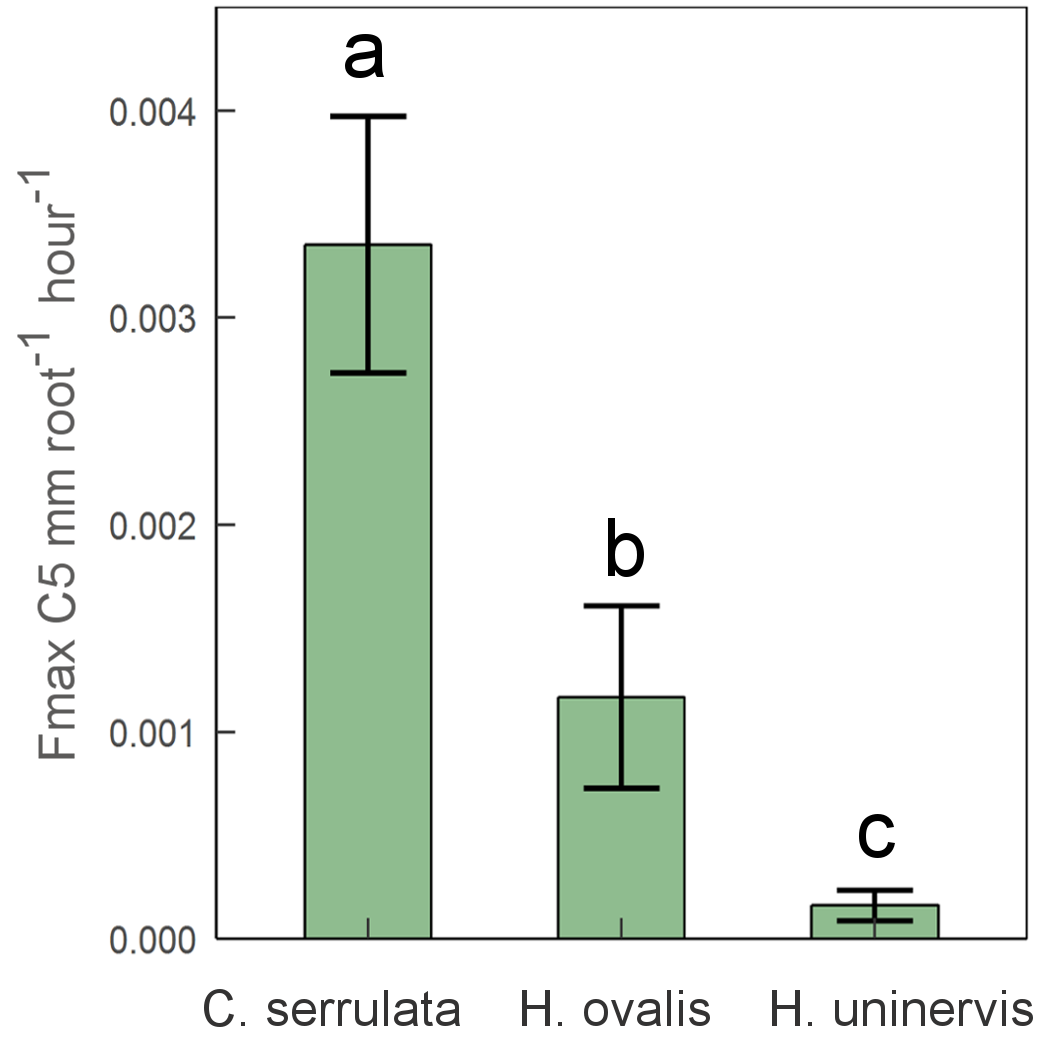
**

**Figure S5.** Root exudation of unclassified fluorescent component C5 for three species of seagrass. Values are means ± 1 SE (n = 12). Significant post-hoc comparisons are indicated with lower case letter.

**Table S6.** Photosynthetic performance of three species of seagrass grown for two weeks under four light treatments, full light control, medium light, low light and fluctuating light (10 days low, four days full light) as assessed using rapid light curves and Pulse Amplitude Modulated (PAM) fluorometry (actinic illumination was incremented in nine steps, each with a duration of 10s). Values are mean (± 1 SD), *n* = 3). Effect of light treatment on individual photosynthetic parameters were investigated using one-way ANOVA for each plant species. Significant post-hoc differences (> 0.05) are indicated by different letters where a significant main effect was found.

| Species | Photosynthetic parameter | Control | Medium | Low | Fluctuating |
| --- | --- | --- | --- | --- | --- |
| *Cymodocea serrulata* | Photosynthetic rate (alpha) | 0.37 (0.07)^A/B^ | 0.48 (0.0.05)^A^ | 0.40 (0.03)^A/B^ | 0.33 (0.03)^B^ |
|  | Maximum photosynthetic capacity (rETRmax) | 56.57 (6.91)^A^ | 55.89 (8.53)^A^ | 43.29 (1.33)^A^ | 80.07 (12.73)^B^ |
|  | Maximum quantum yield (F*_v_*/F*_m_*) | 0.59 (0.10) | 0.68 (0.05) | 0.60 (0.10) | 0.60 (0.0.05) |
| *Halophila ovalis* | Photosynthetic rate (alpha) | 0.40 (0.05) | 0.48 (0.07) | 0.39 (0.02) | 0.44 (0.02) |
|  | Maximum photosynthetic capacity (rETRmax) | 80.70 (10.04) | 75.83 (14.98) | 65.25 (11.74) | 88.95 (45.99) |
|  | Maximum quantum yield (F*_v_*/F*_m_*) | 0.66 (0.06) | 0.64 (0.06) | 0.68 (0.02) | 0.69 (0.04) |
| *Halodule uninervis* | Photosynthetic rate (alpha) | 0.27 (0.02) | 0.35 (0.04) | 0.37 (0.04) | 0.28 (0.03) |
|  | Maximum photosynthetic capacity (rETRmax) | 61.31 (14.30) | 105.95 (42.34) | 94.71 (23.83) | 80.21 (47.34) |
|  | Maximum quantum yield (F*_v_*/F*_m_*) | 0.62 (0.03) | 0.67 (0.07) | 0.73 (0.03) | 0.68 (0.01) |

**References**

Airado-Rodŕiguez, D., Galeano-D́iaz, T., Durán-Merás, I., and Wold, J. P. (2009). Usefulness of fluorescence excitation-emission matrices in combination with PARAFAC, as fingerprints of red wines. *J. Agric. Food Chem.* 57, 1711–1720. doi:10.1021/jf8033623.

Fellman, J. B., Hood, E., and Spencer, R. G. M. (2010). Fluorescence spectroscopy opens new windows into dissolved organic matter dynamics in freshwater ecosystems: A review. *Limnol. Oceanogr.* 55, 2452–2462. doi:10.4319/lo.2010.55.6.2452.

Jørgensen, L., Stedmon, C. A., Kragh, T., Markager, S., Middelboe, M., and Søndergaard, M. (2011). Global trends in the fluorescence characteristics and distribution of marine dissolved organic matter. *Mar. Chem.* 126, 139–148. doi:10.1016/j.marchem.2011.05.002.

Murphy, K. R., Stedmon, C. A., Waite, T. D., and Ruiz, G. M. (2008). Distinguishing between terrestrial and autochthonous organic matter sources in marine environments using fluorescence spectroscopy. *Mar. Chem.* 108, 40–58. doi:10.1016/j.marchem.2007.10.003.

Yamashita, Y., Jaffé, R., Maie, N., and Tanoue, E. (2008). Assessing the dynamics of dissolved organic matter (DOM) in coastal environments by excitation emission matrix fluorescence and parallel factor analysis (EEM-PARAFAC). *Limnol. Oceanogr.* 53, 1900–1908. doi:10.4319/lo.2008.53.5.1900.
